# Comprehensive evaluation of a cost-effective method of culturing *Chlorella pyrenoidosa* with unsterilized piggery wastewater for biofuel production

**DOI:** 10.1186/s13068-019-1407-x

**Published:** 2019-04-01

**Authors:** Weiguo Zhang, Jiangye Li, Zhenhua Zhang, Guangping Fan, Yuchun Ai, Yan Gao, Gang Pan

**Affiliations:** 1 Institute of Agricultural Resources and Environment, Jiangsu Academy of Agricultural Sciences, 50 Zhongling Street, Nanjng, 210014 China; 2 Key Lab of Food Quality and Safety of Jiangsu Province-State Key Laboratory Breeding Base, 50 Zhongling Street, Nanjing, 210014 China; 3 School of Animal Rural and Environmental Sciences, Nottingham Trent University, Brackenhurst, Southwell, Nottinghamshire NG25 0QF UK

**Keywords:** *Chlorella pyrenoidosa*, Biofuel, Unsterilized piggery wastewater, Nutrient removal, Vampirovibrionales

## Abstract

**Background:**

The utilization of *Chlorella* for the dual goals of biofuel production and wastewater nutrient removal is highly attractive. Moreover, this technology combined with flue gas (rich in CO_2_) cleaning is considered to be an effective way of improving biofuel production. However, the sterilization of wastewater is an energy-consuming step. This study aimed to comprehensively evaluate a cost-effective method of culturing *Chlorella pyrenoidosa* in unsterilized piggery wastewater for biofuel production by sparging air or simulated flue gas, including algal biomass production, lipid production, nutrient removal rate and the mutual effects between algae and other microbes.

**Results:**

The average biomass productivity of *C. pyrenoidosa* reached 0.11 g L^−1^ day^−1^/0.15 g L^−1^ day^−1^ and the average lipid productivity reached 19.3 mg L^−1^ day^−1^/30.0 mg L^−1^ day^−1^ when sparging air or simulated flue gas, respectively. This method achieved fairish nutrient removal efficiency with respect to chemical oxygen demand (43.9%/55.1% when sparging air and simulated flue gas, respectively), ammonia (98.7%/100% when sparging air and simulated flue gas, respectively), total nitrogen (38.6%/51.9% when sparging air or simulated flue gas, respectively) and total phosphorus (42.8%/60.5% when sparging air or simulated flue gas, respectively). Culturing *C. pyrenoidosa* strongly influenced the microbial community in piggery wastewater. In particular, culturing *C. pyrenoidosa* enriched the abundance of the obligate parasite Vampirovibrionales, which can result in the death of *Chlorella*.

**Conclusion:**

The study provided a comprehensive evaluation of culturing *C. pyrenoidosa* in unsterilized piggery wastewater for biofuel production. The results indicated that this cost-effective method is feasible but has considerable room for improving. More importantly, this study elucidated the mutual effects between algae and other microbes. In particular, a detrimental effect of the obligate parasite Vampirovibrionales on algal biomass and lipid production was found.

**Electronic supplementary material:**

The online version of this article (10.1186/s13068-019-1407-x) contains supplementary material, which is available to authorized users.

## Background

In the future, humans will face increasingly urgent challenges from the demand for energy. Unfortunately, fossil fuels are not sustainable energy resources. Therefore, the effective solution is to exploit renewable energy resources. At present, in view of their faster growth than other energy crops, microalgae are an ideal alternative to produce biodiesel [1, 2]. The growth of microalgae requires only sunlight, water, CO_2_, and nutrients. It is well known that stock-farming wastewater, municipal wastewater and some industrial wastewaters are rich in nutrients, especially nitrogen (N) and phosphorus (P) [3]. Consequently, the utilization of microalgae for the dual goals of biomass production and wastewater purification is an eco-friendly industry with excellent prospects [2, 4, 5]. The utilization efficiency of CO_2_ in microalgae can reach 20% [6]. Extra CO_2_ supply is believed to be a promising approach for scaled-up algal biomass production [7]. To date, the eco-friendly biotechnology of using flue gas to cultivate microalgae has also been widely explored [8, 9].

*Chlorella* with high carbohydrate or lipid content is an ideal material for biofuel production [10, 11]. Moreover, due to its high tolerance to soluble organic compounds, *Chlorella* is commonly used in wastewater treatment technology [12, 13]. In recent decades, the swine industry has developed rapidly in China, and the number of live swine has been ranked the highest in the world, resulting in serious environmental problems [14]. Piggery/swine wastewater hosts a complex community of microorganisms [15]. Bacterial infection represses the growth of some algae and simultaneously affects the algal cell density and lipid content [16]. Moreover, some bacteria can cause microalgae death by releasing soluble cellulose enzymes [17]. However, the detrimental effects of bacteria on *Chlorella* are unknown. To avoid such unknown detrimental effects, wastewater should be pretreated by sterilizing; however, this is a costly and energy-intensive process, which leads to bottlenecks in scaling up the cultivation of microalgae in piggery wastewaters [18, 19]. To date, there have been a number of studies regarding the technology of culturing *Chlorella* with sterilized piggery/swine wastewater [18–31]. However, little work has been reported on culturing *Chlorella* with unsterilized piggery/swine wastewater for biofuel production [32]. Therefore, the feasibility of culturing *Chlorella* with unsterilized piggery wastewater for biofuel production needs to be further demonstrated. More importantly, the relationship between bacteria and *Chlorella* needs to be clarified urgently. Consequently, this study aimed to comprehensively evaluate a cost-effective way of culturing *Chlorella* with unsterilized piggery wastewater for biofuel production under the condition of sparging air or simulated flue gas, including algal biomass production, lipid production and nutrient removal rate. More importantly, the mutual effects between algae and other microbes were also studied.

## Results and discussion

### Biomass and biofuel production of *C. pyrenoidosa*

**Fig. 1:**
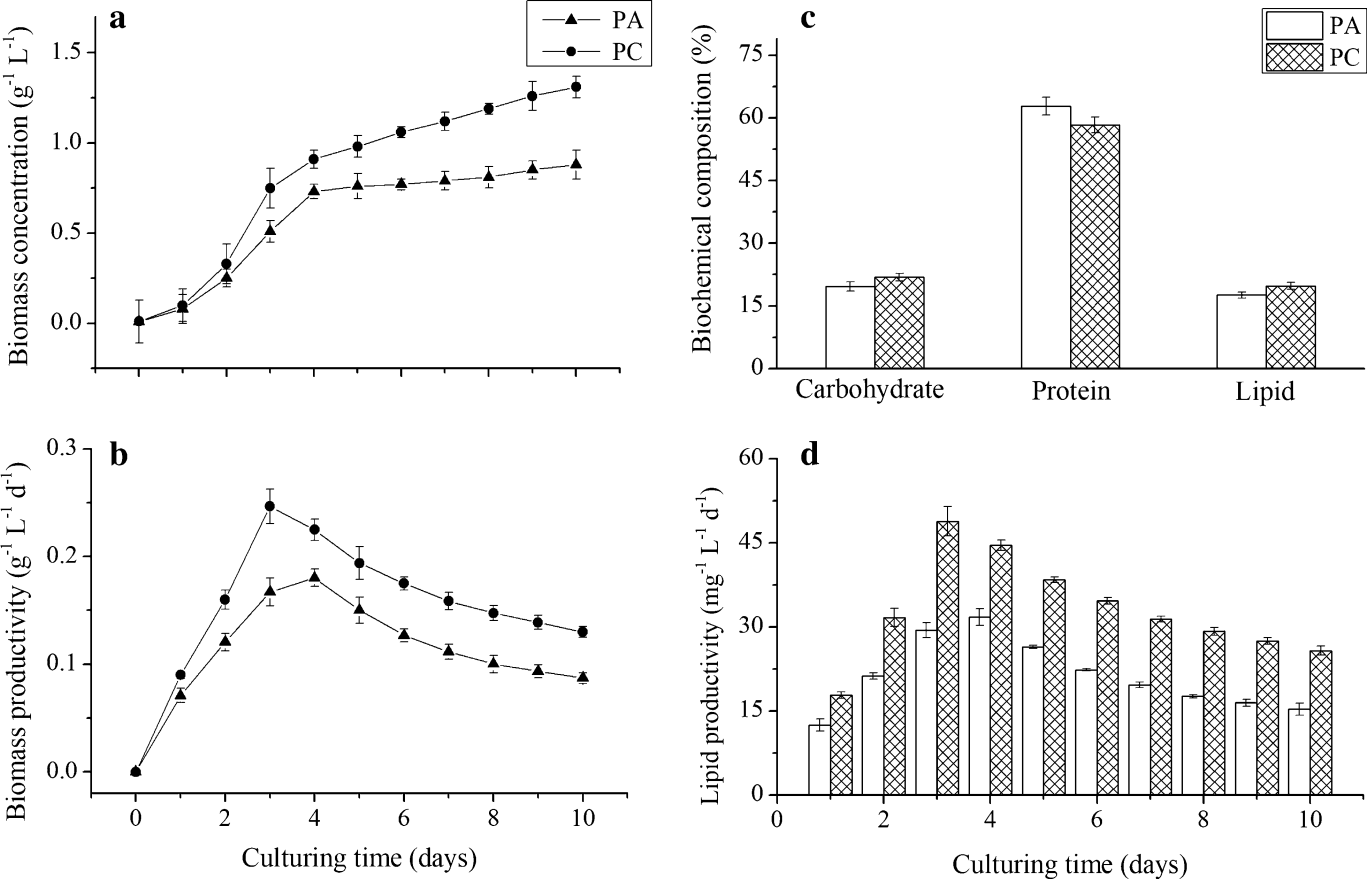
Algal biomass and lipid production of *C. pyrenoidosa* in unsterilized piggery wastewater when sparging air or simulated flue gas. The cultures were illuminated at 28 ± 0.5 °C under a 16-/8-h light/dark cycle with exposure to 45 μE m^−2^ s^−1^ provided by cool-white fluorescent lights. The microalgal cells were sampled every 24 h for growth determinations. PA means culturing *C. pyrenoidosa* with sparging air, and PC means culturing *C. pyrenoidosa* with sparging simulated flue gas. Data are presented as the mean ± standard deviation of the mean

According to the concentration range of nutrients reported in the previous literatures [18–31], the supernatant of piggery wastewater was diluted (1:4) with sterile water before used for culturing microalgae. The concentrations of COD, ammonium, total nitrogen, and total phosphorus in the diluted piggery wastewater were 327.3 mg L^−1^, 11.6 mg L^−1^, 33.7 mg L^−1^ and 7.8 mg L^−1^, respectively. After 10 days, the whole culturing process was finished. The growth potential of *C. pyrenoidosa* sparged with simulated flue gas was higher than that of *C. pyrenoidosa* sparged with air (Fig. 1a). The biomass concentration was 0.88/1.31 g L^−1^, and the specific growth rate (*μ*) was 0.713/0.821 day^−1^ when sparging air or simulated flue gas, respectively. Figure 1b shows the biomass productivity of *C. pyrenoidosa* in unsterilized piggery wastewater when sparging air or simulated flue gas. The average biomass productivity of *C. pyrenoidosa* sparged with simulated flue gas (0.15 g L^−1^ day^−1^) was higher than that of *C. pyrenoidosa* sparged with air (0.11 g L^−1^ day^−1^). Although the lipid content had no significant changes (Fig. 1c), the average lipid productivity when sparging simulated flue gas (30.0 mg L^−1^ day^−1^) was much higher than that under the condition of sparging air (19.3 mg L^−1^ day^−1^) due to the higher algal biomass productivity (Fig. 1d). Sparging flue gas into culture medium is an eco-effective way to increase the algal biomass, lipid content and production [8, 9]. Corresponding to the reported results, both algal biomass and lipid production were increased by sparging flue gas. Extra CO_2_ supply can increase the lipid content of *Chlorella*, possibly because an elevated CO_2_ concentration pushes cells to channel photosynthetic carbon precursors into fatty acid synthesis pathways, resulting in an increase in overall triacylglycerol generation [33]. However, the promoting effects of simulated flue gas on algal lipid content were weak in this study.

The biomass concentration, biomass productivity and lipid productivity of *Chlorella* in piggery wastewater, which varied in different studies, depended on the algal strain, nutrient components/concentration, ratio of C/N/P, pretreatment method, culture condition, etc. [18–32]. Therefore, it is insufficient to evaluate a technology just based on biomass concentration, biomass productivity and lipid productivity. In our study, these parameters had considerable room for improving by optimizing the nutrient components/concentration, nutrient ratio (C/N/P), illumination intensity, aeration mode and so on. Sterilization is indeed a costly and energy-intensive process, which leads to bottlenecks in scaling up the cultivation of microalgae in piggery wastewaters [18, 19]. Consequently, the method of culturing *Chlorella* with unsterilized piggery wastewater for biofuel production should be regarded as a sustainable and cost-effective technology.

### Nutrient removal efficiency

**Fig. 2:**
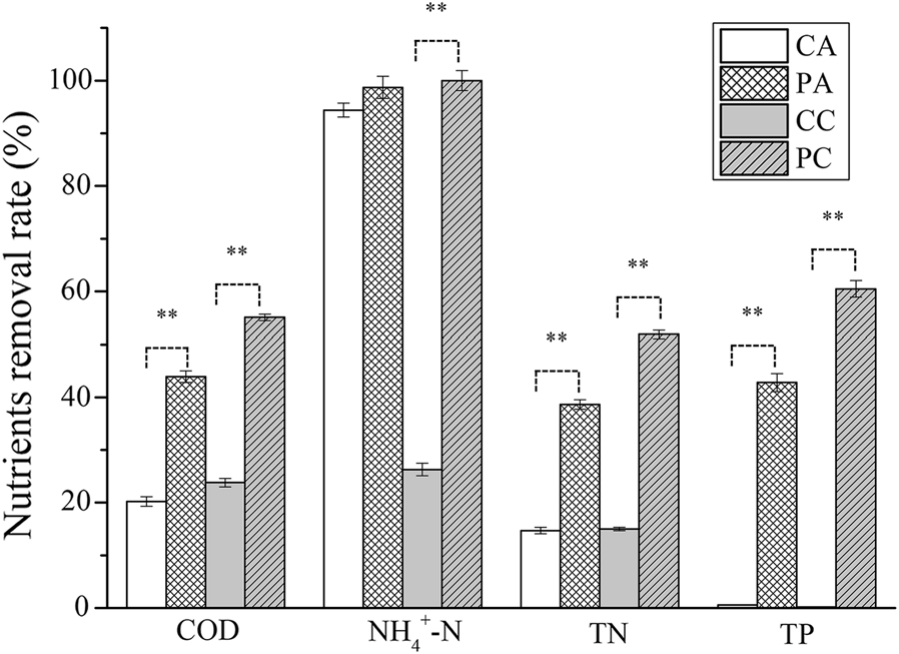
Chemical oxygen demand (COD), ammonium (NH_4_^+^-N), total nitrogen (TN) and total phosphate (TP) removal rates. CA means sparging air, CC sparging simulated flue gas, PA means culturing *C. pyrenoidosa* with sparging air, and PC means culturing *C. pyrenoidosa* with sparging simulated flue gas. Data are presented as the mean ± standard deviation of the mean. **Indicates that there was an extremely significant difference with *P *< 0.01

The nutrient removal efficiencies of culturing *C. pyrenoidosa* in unsterilized piggery wastewater when sparging air or simulated flue gas were studied (Fig. 2). The concentration of COD experienced an obvious decrease when culturing *C. pyrenoidosa* in this study. The removal rate of COD was 43.9%/55.1% when sparging air or simulated flue gas, respectively. When *Chlorella* was cultured in piggery/swine wastewater, the COD removal rate varied in different studies [18–32, 34]. A COD removal rate achieved 99% by reducing ammonia concentration and optimizing C/N ratio (25:1) with culturing *C. vulgaris* after 7-day cultivation in sterilized piggery wastewater, which was the maximum in the current literatures [20]. In this study, the ammonium removal rate of *C. pyrenoidosa* was 98.7% when sparging air and 100% when sparging simulated flue gas, while it fluctuated between 70 and 100% in the reported results [18–32, 34]. The high removal efficiency was due to ammonium being the preferred nitrogen source for most microalgae [35]. In addition, even when *C. pyrenoidosa* was not cultured, the decrease in ammonium concentration was also obvious when sparging air. This should be attributed to the ammoxidation—a biochemical process needing oxygen. Sparging air promoted this biochemical process, resulting in a significant reduction in ammonium. The highest removal rate of TN was also reported by Zheng et al. [20]. The highest removal rate of TP in sterilized piggery wastewater was 98.17% when culturing *C. zofingiensis* [19]. In this study, the TN removal rates of *C. pyrenoidosa* were 38.6% when sparging air and 51.9% when sparging simulated flue gas, while the removal rates of TP were 42.8% when sparging air and 60.5% when sparging simulated flue gas.

### Effects of culturing *C. pyrenoidosa* on bacterial abundance and community

**Fig. 3:**
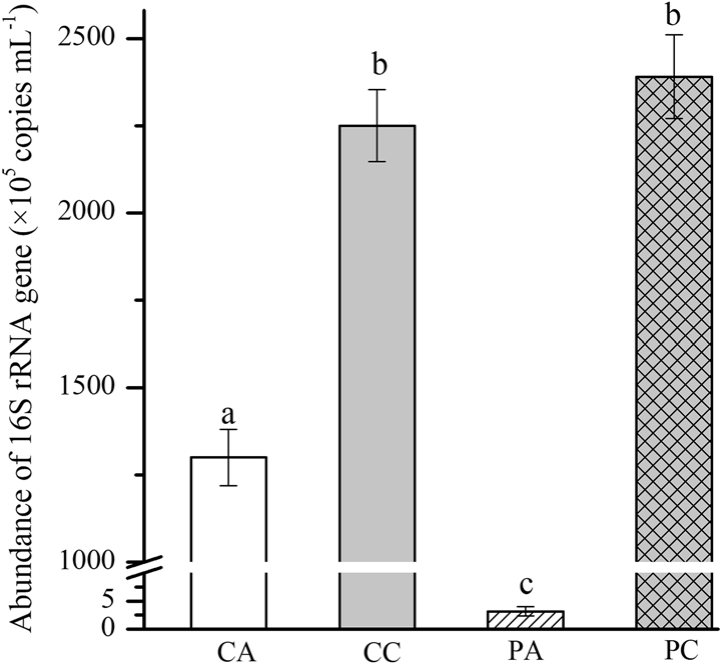
The absolute bacterial abundance based on 16S rRNA copies in unsterilized piggery wastewater. CA means sparging air, CC means sparging simulated flue gas, PA means culturing *C. pyrenoidosa* with sparging air, and PC means culturing *C. pyrenoidosa* with sparging simulated flue gas. Data are presented as the mean ± standard deviation of the mean. The different letters indicate that there was a significant difference with *P *< 0.05

Figure 3 shows the effects of culturing *C. pyrenoidosa* on bacterial abundance in unsterilized piggery wastewater. When sparging air, culturing *C. pyrenoidosa* suppressed the bacterial abundance significantly: the number of 16S rRNA gene copies decreased from 1.3 × 10^8^ copies mL^−1^ (without culturing *C. pyrenoidosa*) to 3.2 × 10^5^ copies mL^−1^ (culturing *C. pyrenoidosa*). However, when sparging simulated flue gas, culturing *C. pyrenoidosa* had no effect on bacterial abundance.

**Fig. 4:**
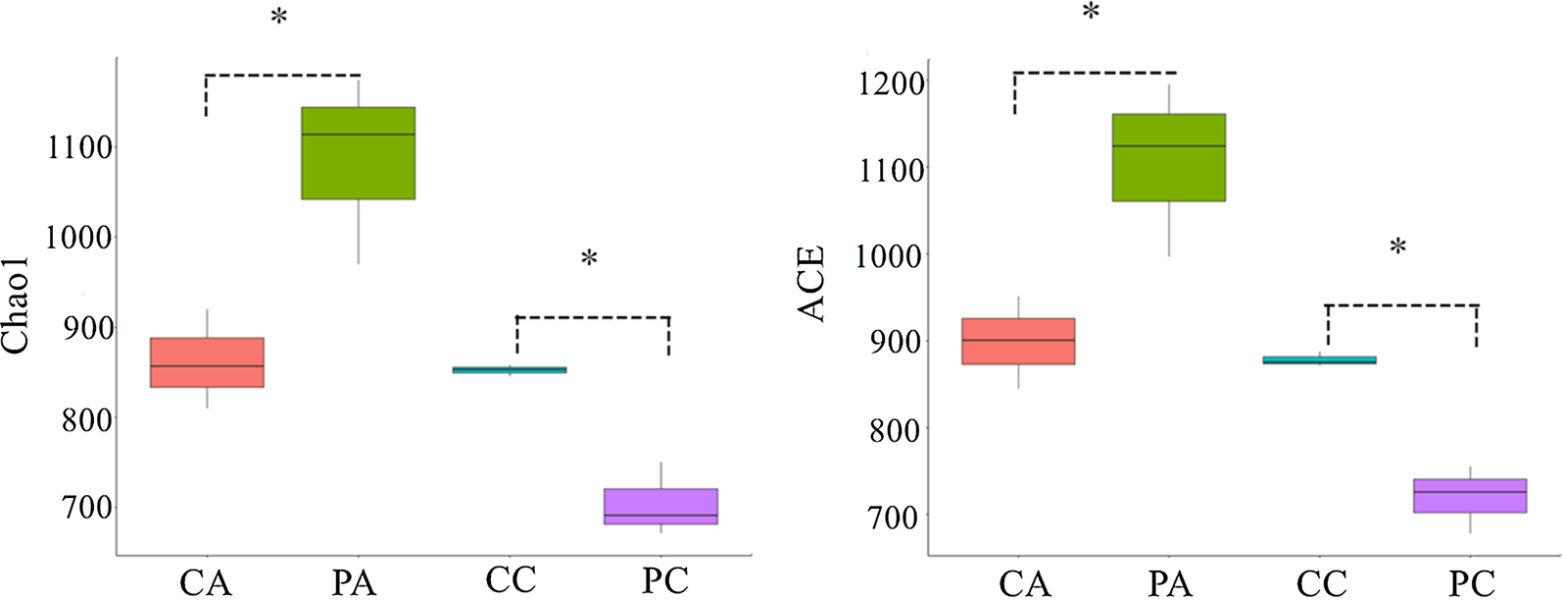
The boxplots of alpha indices (Chao1 and ACE). CA means sparging air, CC sparging simulated flue gas, PA means culturing *C. pyrenoidosa* with sparging air, and PC means culturing *C. pyrenoidosa* with sparging simulated flue gas. *Indicates that there was a significant difference with *P* < 0.05. The statistical test used to compare the indices was the Wilcoxon signed-rank test

The analysis of the bacterial community provided deep insights into the mutual effects between *C. pyrenoidosa* and other microbes. The high-throughput sequencing of 16S rRNA V4 region amplicons yielded 1,073,927 raw reads. After filtering low-quality reads and trimming the adapters, barcodes and primers, there were 1,012,135 valid reads (average length 253 bp). A total of 3185 operational taxonomic units (OTUs) (97% sequence similarity) were clustered. The bacterial abundance was significantly suppressed by culturing *C. pyrenoidosa* under sparging air (Fig. 3), whereas the bacterial diversity was increased (Fig. 4). Under sparging simulated flue gas, culturing *C. pyrenoidosa* decreased the microbial diversity (Fig. 4).

**Fig. 5:**
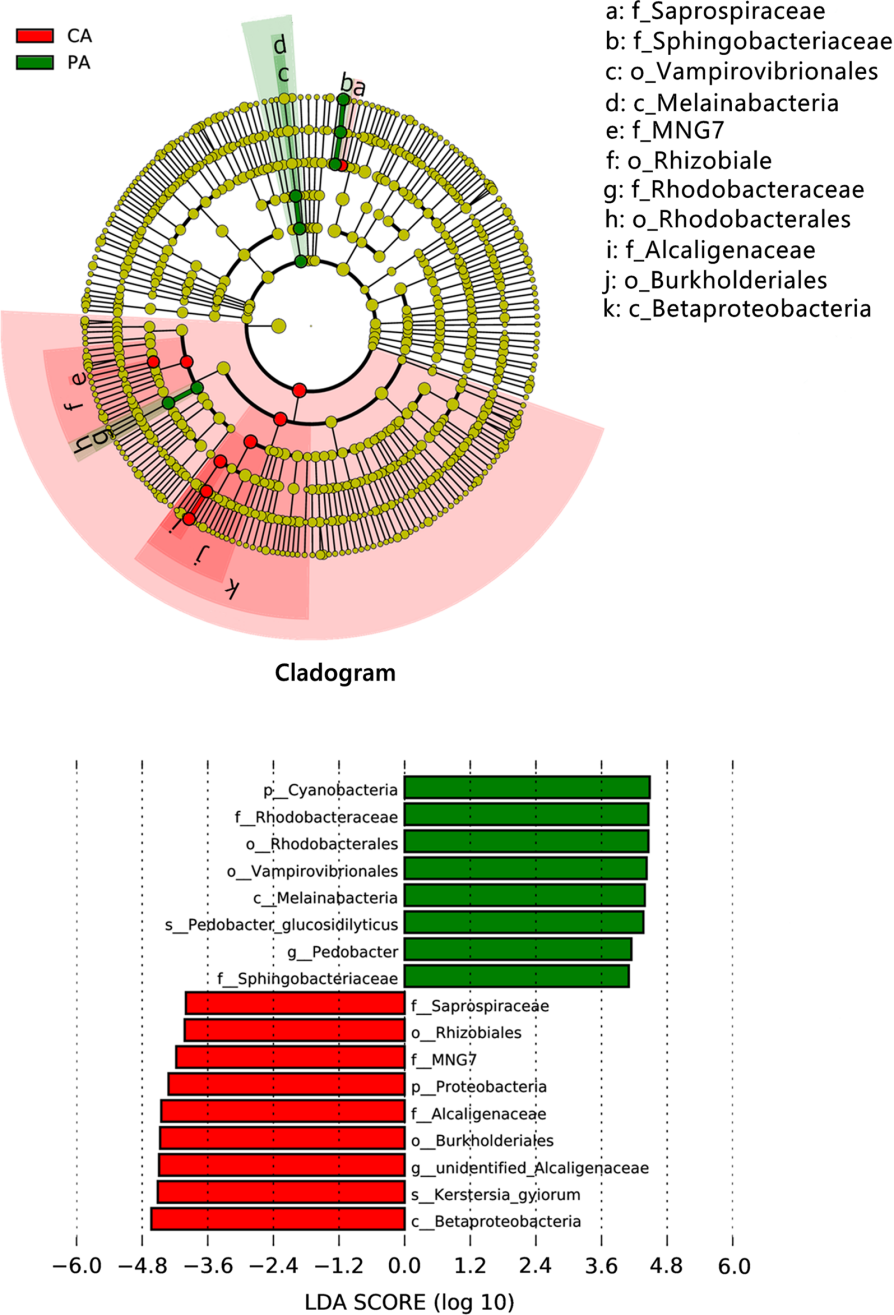
LEfSe analysis identified the most differentially abundant taxa between CA and PA. The taxonomic cladogram was obtained from LEfSe analysis of 16S rRNA sequences; only taxa meeting an LDA significance threshold of 4.0 are shown. Small circles and shading with different colors in the diagram represent the abundance of those taxa in the respective group. Yellow circles represent nonsignificant differences in abundance between CA and PA for that particular taxonomic group. The brightness of each dot is proportional to its effect size. Taxa enriched in PA are shown with a positive LDA score (green) and taxa enriched in CA have a negative score (red). CA means sparging air; PA means culturing *C. pyrenoidosa* with sparging air

**Fig. 6:**
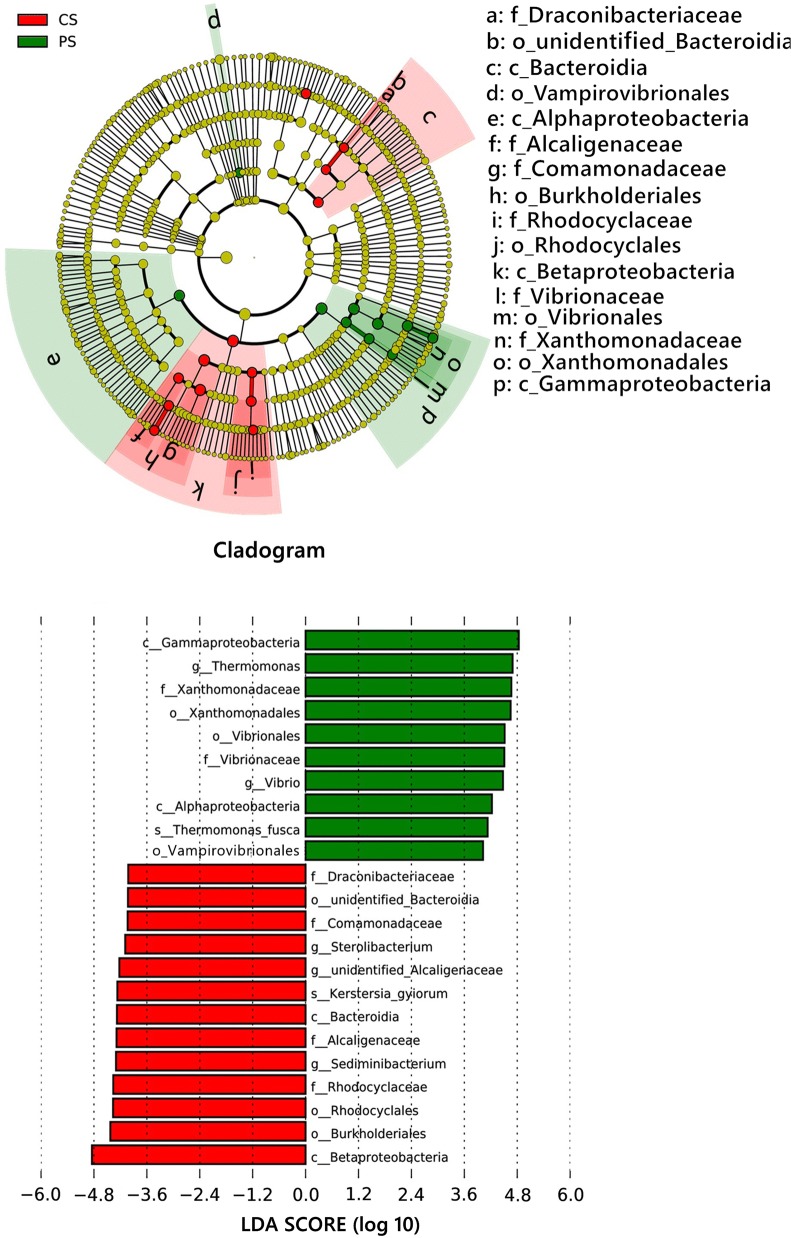
LEfSe analysis identified the most differentially abundant taxa between CC and PC. The taxonomic cladogram was obtained from LEfSe analysis of 16S rRNA sequences; only taxa meeting an LDA significance threshold of 4.0 are shown. Small circles and shading with different colors in the diagram represent the abundance of those taxa in the respective group. Yellow circles represent nonsignificant differences in abundance between CC and PC for that particular taxonomic group. The brightness of each dot is proportional to its effect size. Taxa enriched in PC are shown with a positive LDA score (green), and taxa enriched in CC have a negative score (red). CC means sparging simulated flue gas; PC means culturing *C. pyrenoidosa* with sparging simulated flue gas

LEfSe clearly indicated the effects of culturing *C. pyrenoidosa* on the bacteria (Figs. 5 and 6). Dogs et al. [36] found that Rhodobacteraceae was the predominant family constituting 23% of the epibacterial community of the marine brown algae *Fucus spiralis* and showed physiological adaptation to an epiphytic lifestyle. In this study, Rhodobacteraceae with a relative abundance of 6.8% was also the dominant family in samples culturing *C. pyrenoidosa* when sparging air. It has been reported that the family Rhodobacteraceae is deeply involved in sulfur and carbon biogeochemical cycling [37]. Vampirovibrionales, commonly found in the human gut and groundwater, belongs to a new phylum related to *Cyanobacteria*. The members of Vampirovibrionales are obligate parasites that attach to the cell wall of green alga of *Chlorella* [38], resulting in the death of *Chlorella*. In this study, Vampirovibrionales were the major bacteria, constituting 0.7–6.7% of the bacterial community in the detected samples. It is noteworthy that the Vampirovibrionales were significantly enriched by culturing *C. pyrenoidosa*. Under the condition of sparging air, the abundance of Vampirovibrionales when culturing *C. pyrenoidosa* was 7.6 times as high as that without culturing *C. pyrenoidosa.* Under the condition of sparging simulated flue gas, the abundance of Vampirovibrionales with culturing *C. pyrenoidosa* was 2.7 times as high as that without culturing *C. pyrenoidosa.* The results indicated that *C. pyrenoidosa* suffered from infection by Vampirovibrionales, and this greatly impeded the increase in algal biomass. *Pedobacter glucosidilyticus* was also enriched by culturing *C. pyrenoidosa*, whereas *Kerstersia gyiorum*, *MNG7* and Saprospiraceae were suppressed. *K. gyiorum* is a pathogenic member of the family Alcaligenaceae and is commonly isolated from leg wounds, chronic ear infections, human feces, sputum, and even bronchoalveolar lavage fluids and the urinary tract [39–41]. The suppression of pathogenic microbes by *C. pyrenoidosa* might contribute to a decrease in the risk to public health. The Saprospiraceae, a family within the order Sphingobacteriales, have a demonstrated ability for the hydrolysis and utilization of some complex organic sources [42]. Under the condition of sparging simulated flue gas (Fig. 5), Comamonadaceae, Draconibacteriaceae, Sediminibacterium, Sterolibacterium, and *K. gyiorum* were significantly suppressed by *C. pyrenoidosa.* However, the bacteria of Alphaproteobacteria, Melainabacteria, Vibrio, and *Thermomonas fusca* were enriched by culturing *C. pyrenoidosa*. *Sterolibacterium*, commonly found in anoxic environments, can reduce nitrate to dinitrogen [43].

Clearly, simulated flue gas and culturing *C. pyrenoidosa* both played key roles in structuring the bacterial community. In fact, there are other non-negligible factors that might influence the bacterial community: (1) Algae can excrete a variety of organic compounds, such as carbohydrates, lipopolysaccharides, organohalogens, amino acids and peptides, which are available to many bacteria [44]. In this study, some organic matter originating from *C. pyrenoidosa* could be utilized by specific bacteria during cultivation. However, some studies have indicated that some organic matter of *Chlorella* has antibacterial activity against specific bacteria [45]. Therefore, it is probable that some bacteria in piggery wastewater were inhibited by culturing *C. pyrenoidosa*. (2) The growth of *C. pyrenoidosa* had little effect on pH when sparging simulated flue gas in this study, but the pH (> 8.0) was increased by *C. pyrenoidosa* when sparging air (Additional file 1: Fig. S1). When phytoplankton grows in excessive abundance, photosynthesis by algae during daylight releases oxygen and removes carbon dioxide from the water, resulting in an increase in pH [46, 47]. Consequently, pH influences the bacterial community. (3) Nutrient competition can also influence the relationship between microalgae and bacteria [48, 49]. In this study, the concentrations of ammonium, TN and TP decreased due to culturing *C. pyrenoidosa*, which might also lead to changes in the bacterial community.

The most noteworthy result was that the obligate parasites Vampirovibrionales were significantly enriched by culturing *C. pyrenoidosa*. The bacterium has very specific requirements for growth—it seems to grow only by attachment to the cell wall of intact *Chlorella* cells and consuming their cytoplasmic contents [38, 50]. Although it needs to be further clarified whether the obligate parasites Vampirovibrionales are commonly found in other wastewaters, this result emphasizes the need to adequately consider these obligate parasites when using unsterilized wastewater for culturing *Chlorella*. In other words, the obligate parasite Vampirovibrionales in this study was a restrictive factor in algal growth, lipid accumulation and nutrient removal. More importantly, this result indicates that the selection of algal strain must be carefully performed.

## Conclusion

In this study, we comprehensively evaluated a cost-effective method of using unsterilized piggery wastewater for biofuel production by culturing *Chlorella*. This method achieved moderate algal biomass productivity, lipid productivity and fairish nutrient removal efficiency. Moreover, our results indicated that culturing *C. pyrenoidosa* strongly influenced the microbial community in piggery wastewater. In particular, a detrimental effect of the obligate parasite Vampirovibrionales on algal biomass and lipid production was found.

## Methods

### Piggery wastewater used as culture media

The piggery wastewater used in this study was from a local pig farm, was directly discharged and was stored in a cement pond. The collected wastewater was allowed to settle for 1 day to precipitate. The supernatant was diluted (1:4) with sterile water before being used for culturing microalgae. The concentrations of COD, ammonium, total nitrogen, and total phosphorus in the piggery wastewater were determined following the protocols described previously [51], and the parameters of the original piggery wastewater are shown in Additional file 2: Table S1.

### Algal strain and culture conditions

*C. pyrenoidosa*, a species of *Chlorella,* can tolerate a high concentration of soluble organic compounds and effectively utilize a variety of organic carbon sources in wastewater [52, 53]. Therefore, *C. pyrenoidosa* was selected as a target strain. The green algae *C. pyrenoidosa* was obtained from the Institute of Hydrobiology, Chinese Academy of Sciences (FACHB-10), and grown in BBM medium containing the following composition (per liter): 0.25 g NaNO_3_, 0.075 g K_2_HPO_4_, 0.075 g MgSO_4_·7H_2_O, 0.025 g CaCl_2_·2H_2_O, 0.175 g KH_2_PO_4_, 0.025 g NaCl, 0.75 mg Na_2_-EDTA, 0.097 mg FeCl_3_·6H_2_O, 1 mg vitamin B_1_, 0.25 μg biotin, 0.15 μg vitamin B_12_, 0.041 mg MnCl_2_·4H_2_O, 0.005 mg ZnCl_2_·7H_2_O, 0.004 mg Na_2_MoO_4_·2H_2_O and 0.002 mg CoCl_2_·6H_2_O. The algal cells were axenically grown at 28 ± 0.5 °C under a 16-/8-h light/dark cycle with exposure to 45 μE m^−2^ s^−1^ provided by cool-white fluorescent lights. The cool-white fluorescent lights were 0.2 m above the culture flask. After adjusting the pH to 7.0, 500 mL of the pretreated piggery wastewater was placed in a 2000-mL conical flask. *C. pyrenoidosa* in the linear growth phase was used as the inoculum. The initial inoculation density was 2 × 10^6^ cells mL^−1^. The culture medium without mechanical oscillation was sparged with sterilized air or simulated flue gas (CO_2_ 20%, N_2_ 80%) at a flow rate of 0.5 L min^−1^. The experiments were divided into four groups: sparging air (CA), sparging air with culturing *C. pyrenoidosa* (PA), sparging simulated flue gas (CC) and sparging CO_2_ with culturing *C. pyrenoidosa* (PC). All experiments were conducted in triplicate.

### Growth of *C. pyrenoidosa*

The growth of *C. pyrenoidosa* was determined by measuring the total chlorophyll concentration (∑C) using a spectrophotometric method [18, 54]. The biomass concentration (dry weight of cell powder (DCW) in culture medium, g L^−1^) in the piggery wastewater was estimated by an equation that employs the total chlorophyll (∑C):

1 $$ {\text{DCW }}\left( {{\text{g L}}^{ - 1} } \right) \, = \, 0.1084\sum C, \, R^{2} = \, 0.9562 $$

The specific growth rate (μ) was calculated by fitting the total chlorophyll in the exponential phase of algal growth, which was measured by the following formula:

2 $$ \mu \left( {{\text{day}}^{ - 1} } \right) \, = \, {{\left( {{ \ln }\sum C - { \ln }\sum C_{0} } \right)} \mathord{\left/ {\vphantom {{\left( {{ \ln }\sum C - { \ln }\sum C_{0} } \right)} t}} \right. \kern-0pt} t}, $$

where *t* (day) is the time between two measurements and *∑C* and *∑C*_*0*_ (mg L^−1^) are the total chlorophyll concentrations at the start and end of the exponential phase, respectively. The biomass productivity (*P*) was calculated according to the following formula [7]:

3 $$ P \, = \, \left( {dw_{i} - dw_{o} } \right)/\left( {t_{i} - t_{0} } \right), $$

where *dw*_*i*_ and *dw*_*o*_ are dry biomass (g L^−1^) at time *t*_*i*_ and *t*_*0*_ (initial time), respectively.

### Determination of lipid, protein and carbohydrate content and productivity

The biochemical composition of algae was determined by Fourier transform infrared (FTIR) spectrometry. The FTIR analysis was performed as previously described by Zhang et al. [53]. Briefly, cell pellets centrifuged at 8000*g* for 10 min were washed twice with deionized water. Deionized water was used to resuspend the cell pellets at a concentration of approximately 1.0 mg mL^−1^ (dry weight). A vacuum drying oven was used to dry a total of 200-μL suspension, which was dropped on a KRS-5 window (30 × 5 mm) at 40 °C. The transmittance spectra were collected between 400 and 4000 cm ^−1^ at a resolution of 4 cm^−1^ with 32 scans on an FTIR spectrometer (NEXUS 870, Thermo Nicolet, USA). The data were processed with OMNIC 6.0 software. The spectrum baseline was corrected by a rubber-band method using 64 baseline points with the exclusion of CO_2_ bands.

The characteristic peak areas of lipids (*A*_*L*_), proteins (*A*_*P*_) and carbohydrates (*A*_*C*_) were calculated by integration. The weights (mg) of lipids (*W*_*L*_), proteins (*W*_*P*_) and carbohydrates (*W*_*C*_) were calculated according to the following formulas [55]:

4 $$ A_{L} = \, -\, 2. 30 + { 78}. 9 6\times W_{L} $$

5 $$ A_{p} = \, - \,0. 2 7+ { 12}. 7 2\times W_{p} $$

6 $$ A_{p} = \, -\, 0. 2 7+ { 12}. 7 2\times W_{p} $$

Assuming that the algal cells consisted of only lipids, proteins and carbohydrates, the contents (%) of lipids (*C*_*L*_), proteins (*C*_*P*_*)* and carbohydrates (*C*_*C*_) were calculated with the following formulas [56]:

7 $$ C_{L} = \,\hbox{W}_{\rm L}/ (\hbox{W}_{\rm L} + \hbox{W}_{\rm P} + \hbox{W}_{\rm C})\times 100 $$

8 $$ C_{P} = \,\hbox{W}_{\rm P}/ (\hbox{W}_{\rm L} + \hbox{W}_{\rm P} + \hbox{W}_{\rm C})\times 100 $$

9 $$ C_{C} = \,\hbox{W}_{\rm C}/ (\hbox{W}_{\rm L} + \hbox{W}_{\rm P} + \hbox{W}_{\rm C})\times 100 $$

The lipid productivity (*P*_*L*_) was calculated according to the following formula:

10 $$ P_{L} = \,(dw_i-dw_o) \times C_L/((t_i-t_0) \times 100), $$

where *dw*_*i*_ and *dw*_*o*_ are the dry biomass (g L^−1^) at times *t*_*i*_ and *t*_*0*_ (initial time), respectively. *C*_*L*_ is the lipid content (%).

### Sampling and nutrient analysis

A volume of 5-mL microalgae suspension was collected every day from each conical flask in a clean bench for nutrient analysis starting from inoculation. The samples were first centrifuged at 5000 rpm for 10 min, after which the supernatants were filtered using a 0.22-μm nylon membrane filter. Then, the filtrates were appropriately diluted and analyzed for ammonia, total nitrogen, and total phosphate following the Hach DR 2700 Spectrophotometer Manual. The nutrient removal rate was obtained using the following expression [19]:

11 $$ Nutrient  \ removal \ rate \  W \%  = \,100 \% \times (C_0-C_i)/C_0, $$

where *C*_*o*_ and *C*_*i*_ are defined as the mean nutrient concentrations at the initial time *t*_*0*_ and time *t*_*i*_, respectively.

### DNA extraction and sequencing library construction

After the *C. pyrenoidosa* grew for 10 days, the medium was oscillated at a speed of 100 r min^−1^, and then 0.05-L samples from each flask were filtered with 0.22-μm filter membranes using a filtration apparatus. The obtained membranes were stored at − 80 °C until DNA extraction. Before DNA extraction, all the filter membranes were cut into pieces with sterile scissors. DNA extraction was performed using an E.Z.N.A. Water DNA Kit (OMEGA Bio-Tek Inc., USA) according to the manufacturer’s instructions. The extracted DNA was stored in a freezer at − 80 °C prior to downstream analysis. The 16S rRNA amplicons were amplified by primer pair 515F/806R (515F: 5′-NNNNNNNNGTGTGCCAGCMGCCGCGGTAA-3′, 806R: 5′-GGACTACHVGGGTWTCTAAT-3′) targeting the V4 hypervariable region of 16S rRNA genes [57]. The high-throughput sequencing of 16S rRNA amplicons was performed on the Illumina MiSeq platform at Novogene Bioinformatics Company (Beijing, China).

### Sequencing data analysis

Paired-end reads were assigned based on the unique barcodes of samples, which were subsequently truncated by cutting off the barcode and primer sequence. The paired-end reads were merged using FLASH (V1.2.7) into raw tags. Quality filtering on the raw tags was performed to obtain high-quality clean tags according to QIIME (V1.7.0). The tags were compared with the reference database (Gold database) using a UCHIME algorithm to detect chimera sequences. The chimera sequences were removed to obtain the effective tags. Sequence analyses were performed using Uparse software (Uparse v7.0.1001). Sequences with ≥ 97% similarity were assigned to the same OTUs. The representative sequence for each OTU was screened for further annotation. For each representative sequence, the GreenGene Database was used based on an RDP classifier (Version 2.2) algorithm to annotate taxonomic information. Alpha diversity indices (Chao1 and ACE) were applied to analyze bacterial diversity. All these indices were calculated with QIIME (Version 1.7.0) and displayed with boxplots drawn by R software (Version 2.15.3).

### Data analysis

With regard to the nutrients remove rate and the bacterial abundance, statistical significance was assessed by analysis of variance (ANOVA) followed by Fisher’s post hoc test using the IBM SPSS Statistics 21.0 program (IBM, Armonk, New York, USA); while, the statistical test used to compare the indices of microbial diversities was the Wilcoxon signed-rank test. A *P* value of less than 0.05 was considered as statistically significant.

## Additional files

**Additional file 1: Figure S1.** The pH in piggery wastewater during the process of culturing *C. pyrenoidosa*. Data are presented as the means ± standard deviation of the mean. CA means sparging air, CC means sparging simulated flue gas, PA means culturing *C. pyrenoidosa* with sparging air, and PC means culturing *C. pyrenoidosa* with sparging simulated flue gas.

**Additional file 2: Table S1.** The pH in piggery wastewater during the process of culturing *C. pyrenoidosa*. Data are presented as the means ± standard deviation of the mean. CA means sparging air, CC means sparging simulated flue gas, PA means culturing *C. pyrenoidosa* with sparging air, and PC means culturing *C. pyrenoidosa* with sparging simulated flue gas.

## References

[1] Chen H, Zheng Y, Zhan J, He C, Wang Q (2017). Comparative metabolic profiling of the lipid-producing green microalga *Chlorella*, reveals that nitrogen and carbon metabolic pathways contribute to lipid metabolism. Biotechnol Biofuels.

[2] Zaimes GG, Khanna V (2013). Microalgal biomass production pathways: evaluation of life cycle environmental impacts. Biotechnol Biofuels.

[3] Chiu SY, Kao CY, Chen TY, Chang YB, Kuo CM, Lin CS (2015). Cultivation of microalgal *Chlorella* for biomass and lipid production using wastewater as nutrient resource. Bioresour Technol.

[4] Toyama T, Kasuya M, Hanaoka T, Kobayashi N, Tanaka Y, Inoue D (2018). Growth promotion of three microalgae, *Chlamydomonas reinhardtii*, *Chlorella vulgaris*, and *Euglena gracilis*, by in situ indigenous bacteria in wastewater effluent. Biotechnol Biofuels.

[5] Jiang L, Zhang L, Nie C, Pei H (2018). Lipid productivity in limnetic *Chlorella*, is doubled by seawater added with anaerobically digested effluent from kitchen waste. Biotechnol Biofuels.

[6] Solovchenko A, Khozin-Goldberg I (2013). High dose CO_2_ tolerance in microalgae: possible mechanisms and implications for biotechnology and bioremediation. Biotechnol Lett.

[7] Ge Y, Liu J, Tian G (2011). Growth characteristics of *Botryococcus braunii* 765 under high CO_2_ concentration in photobioreactor. Bioresour Technol.

[8] Taştan BE, Tekinay T (2016). A novel coal additive from microalgae produced from thermal power plant flue gas. J Clean Prod..

[9] Monari C, Righi S, Olsen SI (2016). Greenhouse gas emissions and energy balance of biodiesel production from microalgae cultivated in photobioreactors in Denmark: a life-cycle modeling. J Clean Prod..

[10] Yeh KL, Chang JS (2012). Effects of cultivation conditions and media composition on cell growth and lipid productivity of indigenous microalga *Chlorella vulgaris* ESP-31. Bioresour Technol.

[11] Ho SH, Huang SW, Chen CY, Hasunuma T, Kondo A, Chang JS (2013). Characterization and optimization of carbohydrate production from an indigenous microalga *Chlorella vulgaris* FSP-E. Bioresour Technol.

[12] de la Noue J, Basseres A (1989). Biotreatment of anaerobically digested swine manure with microalgae. Biol Wastes..

[13] Metting FB (1996). Biodiversity and application of microalgae. J Ind Microbiol Biotechnol.

[14] Huang D, Ma Q, Feng L, Wen X, Li H (2018). Applying data mining to china’s swine farming industry: a compromise perspective of economic, environmental and overall performances. Sustainability..

[15] Desneux J, Pourcher AM (2014). Comparison of DNA extraction kits and modification of DNA elution procedure for the quantitation of subdominant bacteria from piggery effluents with real-time PCR. MicrobiologyOpen.

[16] Zhang Y, Su H, Zhong Y, Zhang C, Shen Z, Sang W, Yan G, Zhou X (2012). The effect of bacterial contamination on the heterotrophic cultivation of *Chlorella pyrenoidosa* in wastewater from the production of soybean products. Water Res.

[17] Kim JD, Kim B, Lee CG (2007). Alga-lytic activity of *Pseudomonas* fluorescens against the red tide causing marine alga *Heterosigma akashiwo* (Raphidophyceae). Biol Control.

[18] Gan K, Mou X, Xu Y, Wang H (2014). Application of ozonated piggery wastewater for cultivation of oil-rich *Chlorella pyrenoidosa*. Bioresour Technol.

[19] Zhu L, Wang Z, Takala J, Hiltunen E, Qin L, Xu Z, Qin X, Yuan Z (2013). Scaleup potential of cultivating *Chlorella zofingiensis* in piggery wastewater for biodiesel production. Bioresour Technol.

[20] Zheng HL, Wu XD, Zou GY, Zhou T, Liu YH, Ruan R (2019). Cultivation of *Chlorella vulgaris* in manure-free piggery wastewater with high-strength ammonium for nutrients removal and biomass production: effect of ammonium concentration, carbon/nitrogen ratio and pH. Bioresour Technol.

[21] Kuo CM, Chen TY, Lin TH, Kao CY, Lai JT, Chang JS, Lin CS (2015). Cultivation of *Chlorella* sp. *GD* using piggery wastewater for biomass and lipid production. Bioresour Technol..

[22] Ji MK, Kim HC, Sapireddy VR, Yun HS, Abou-Shanab RA, Choi J, Lee W, Timmes TC, Jeon BH (2012). Simultaneous nutrient removal and lipid production from pretreated piggery wastewater by *Chlorella vulgaris* YSW-04. Appl Microbiol Biotechnol..

[23] Wang H, Xiong H, Hui Z, Zeng X (2012). Mixotrophic cultivation of *Chlorella pyrenoidosa* with diluted primary piggery wastewater to produce lipids. Bioresour Technol.

[24] Cao L, Zhou T, Li Z, Wang J, Tang J, Ruan R, Liu YH (2018). Effect of combining adsorption-stripping treatment with acidification on the growth of *Chlorella vulgaris* and nutrient removal from swine wastewater. Bioresour Technol.

[25] Wen Y, He Y, Ji X, Li S, Chen L, Zhou Y, Wang MZ, Chen BL (2017). Isolation of an indigenous, *Chlorella vulgaris*, from swine wastewater and characterization of its nutrient removal ability in undiluted sewage. Bioresour Technol.

[26] Cheng PF, Wang YZ, Liu TH, Liu DF (2017). Biofilm attached cultivation of *Chlorella pyrenoidosa* is a developed system for swine wastewater treatment and lipid production. Front Plant Sci..

[27] Nam K, Lee H, Heo SW, Chang YK, Han JI (2017). Cultivation of *Chlorella vulgaris* with swine wastewater and potential for algal biodiesel production. J Appl Phycol.

[28] Amini H, Wang L, Shahbazi A (2016). Effects of harvesting cell density, medium depth and environmental factors on biomass and lipid productivities of *Chlorella vulgaris* grown in swine wastewater. Chem Eng Sci.

[29] Wang Y, Guo W, Yen HW, Ho SH, Lo YC, Cheng CL, Ren NQ, Chang JS (2015). Cultivation of *Chlorella vulgaris* jsc-6 with swine wastewater for simultaneous nutrient/cod removal and carbohydrate production. Bioresour Technol.

[30] Zhang B, Chen S (2015). Effect of different organic matters on flocculation of *Chlorella sorokiniana* and optimization of flocculation conditions in swine manure wastewater. Bioresour Technol.

[31] Mezzari MP, da Silva MLB, Pirolli M, Perazzoli S, Steinmetz RLR, Nunes EO, Soares HM (2014). Assessment of a tannin-based organic polymer to harvest *Chlorella vulgaris* biomass from swine wastewater digestate phycoremediation. Water Sci Technol.

[32] Marjakangas JM, Chen CY, Lakaniemi AM, Puhakka JA, Whang LM, Chang JS (2015). Simultaneous nutrient removal and lipid production with *Chlorella vulgaris* on sterilized and non-sterilized anaerobically pretreated piggery wastewater. Biochem Eng J.

[33] Sun Z, Chen YF, Du J (2015). Elevated CO_2_ improves lipid accumulation by increasing carbon metabolism in *Chlorella sorokiniana*. Plant Biotechnol J.

[34] Wang M, Yang Y, Chen Z, Chen Y, Wen Y, Chen B (2016). Removal of nutrients from undiluted anaerobically treated piggery wastewater by improved microalgae. Bioresour Technol.

[35] Razzak SA, Hossain MM, Lucky RA, Bassi AS, Lasa HD (2013). Integrated CO_2_, capture, wastewater treatment and biofuel production by microalgae culturing-a review. Renew Sust Energ Rev..

[36] Dogs M, Wemheuer B, Wolter L, Bergen N, Daniel R, Simon M, Brinkhoff T (2017). *Rhodobacteraceae* on the marine brown alga *Fucus spiralis* are abundant and show physiological adaptation to an epiphytic lifestyle. Syst Appl Microboil..

[37] Pujalte MJ, Lucena T, Ruvira MA, Arahal DR, Macián MC (2014). The Family Rhodobacteraceae.

[38] Coder DM, Goff LJ (1986). The host range of the Chlorellavorous bacterium (“*Vampirovibrio chlorellavorus*”). J Appl Phycol.

[39] Deutscher M, Severing J, Baladallasat JM (2014). *Kerstersia gyiorum* isolated from a bronchoalveolar lavage in a patient with a chronic tracheostomy. Case Rep Infect Dis..

[40] Ogawa Y, Lee ST, Kasahara K, Koizumi A, Chihara Y, Nakano R, Hisakazuet Y, Mikasa K (2016). A first case of isolation of *Kerstersia gyiorum* from urinary tract. J Infect Chemother..

[41] Lan Y, Yan Q, Yan Y, Liu W (2017). First case of *Kerstersia gyiorum* isolated from a patient with chronic osteomyelitis in China. Front Lab Med..

[42] Mcilroy SJ, Nielsen PH (2014). The prokaryotes.

[43] Tarlera S (2003). Sterolibacterium denitrificans gen. nov., sp. nov., a novel cholesterol-oxidizing, denitrifying member of the-Proteobacteria. Int J Syst Evol Microbiol..

[44] Graham LE, Wilcox LW (2000). Algae.

[45] Alwathnani H, Perveen K (2017). Antibacterial activity and morphological changes in human pathogenic bacteria caused by *Chlorella vulgaris* extracts. Biomed Res-India..

[46] Szyper JP, Ebeling JM (1993). Photosynthesis and community respiration at three depths during a period of stable phytoplankton stock in a eutrophic brackish water culture pond. Mar Ecol Prog Ser.

[47] Gao Y, Zhang Z, Liu XH, Yi N, Zhang L, Song W (2016). Seasonal and diurnal dynamics of physicochemical parameters and gas production in vertical water column of a eutrophic pond. Ecol Eng.

[48] Unnithan VV, Unc A, Smith GB (2014). Mini-review: a priori considerations for bacteria-algae interactions in algal biofuel systems receiving municipal wastewaters. Algal Res.

[49] Ramanan R, Kim BH, Cho DH, Oh HM, Kim HS (2016). Algae-bacteria interactions: evolution, ecology and emerging applications. Biotechnol Adv.

[50] Gromov BV, Mamkaeva KA (1972). Electron microscopic study of parasitism by *Bdellovibrio chlorellavorus* bacteria on cells of the green alga *Chlorella vulgaris*. Tsitologiia..

[51] Clesceri LS, Greenberg AE, Eaton AD. Standard methods for the examination of water and wastewater. 20th ed. American Public Health Association; 1998.

[52] Zhang W, Zhang P, Sun H, Chen M, Lu S, Li P (2014). Effects of various organic carbon sources on the growth and biochemical composition of *Chlorella pyrenoidosa*. Bioresour Technol.

[53] Zhang W, Zhang Z, Yan S (2015). Effects of various amino acids as organic nitrogen sources on the growth and biochemical composition of *Chlorella pyrenoidosa*. Bioresour Technol.

[54] Becker EW, Becker EW (1994). Measurement of algal growth. Microalgae: biotechnology and microbiology.

[55] Pistorius AMA, DeGrip WJ, Egorova-Zachernyu TA (2009). Monitoring of biomass composition from microbiological sources by means of FT-IR spectroscopy. Biotechnol Bioeng.

[56] Feng GD, Zhang F, Cheng LH, Xu XH, Zhang L, Chen HL (2013). Evaluation of FT-IR and Nile Red methods for microalgal lipid characterization and biomass composition determination. Bioresour Technol.

[57] Caporaso JG, Lauber CL, Walters WA, Berg-Lyons D, Lozupone CA, Turnbaugh PJ, Noah FN, Knight R (2011). Global patterns of 16S rRNA diversity at a depth of millions of sequences per sample. Proc Natl Acad Sci USA.

