# Supplementary material for: Comprehensive evaluation of a cost-effective method of culturing Chlorella pyrenoidosa with unsterilized piggery wastewater for biofuel production
Source: Biotechnol Biofuels. 2019 Apr 1;12:69. doi: 10.1186/s13068-019-1407-x (PMC6442423; doi:10.1186/s13068-019-1407-x)
Supplement: Supplementary file 2 — Additional file 2: Table S1. The pH in piggery wastewater during the process of culturing C. pyrenoidosa. Data are presented as the means ± standard deviation of the mean. CA means sparging air, CC means sparging simulated flue gas, PA means culturing C. pyrenoidosa with sparging air, and PC means culturing C. pyrenoidosa with sparging simulated flue gas. [file 13068_2019_1407_MOESM2_ESM.docx]

| Parameters Original piggery wastewater |
| --- |
| pH 7.6±0.2  Total Nitrogen (mg L^−1^) 134.8±5.1  NH_4_^+^-N (mg L^−1^) 46.2±1.8  Total Phosphate (mg L^−1^) 31.3±1.5  COD (mg L^−1^) 1309±17.3 |
